# Are People More Inclined to Vote at 16 than at 18? Evidence for the First-Time Voting Boost Among 16- to 25-Year-Olds in Austria

**DOI:** 10.1080/17457289.2013.872652

**Published:** 2014-01-08

**Authors:** Eva Zeglovits, Julian Aichholzer

**Affiliations:** ^a^ University of Vienna, Austria

## Abstract

Potential consequences of lowering voting age to 16 have been discussed in recent scientific and public debates. This article examines turnout of young voters aged 16 to 17 in Austria, the first European country that lowered the general voting age to 16. For this purpose we use unique data taken from electoral lists of two recent Austrian regional elections. The results support the idea that the so-called “first-time voting boost” is even stronger among the youngest voters as turnout was (a) higher compared to 18- to 20-year-old first-time voters and (b) not substantially lower than the average turnout rate. We conclude that our findings are encouraging for the idea of lowering voting age as a means to establish higher turnout rates in the future.

## 1. Introduction

There is overwhelming evidence from various countries that electoral turnout among young voters is significantly and substantially lower than in the overall electorate (e.g. Arzheimer, 2006; Bhatti & Hansen, 2012b; Blais et al., 2004; Blais & Rubenson, 2013; Electoral Commission, 2002; Fieldhouse et al., 2007; Gallego, 2009; Milner, 2009; Rubenson et al., 2004; Topf, 1995; Wass, 2007; Wattenberg, 2002, 2008).*This article makes reference to supplementary material available on the publisher's website at http://dx.doi.org/10.1080/17457289.2013.872652
 More precisely, the relation between turnout and age is a curvilinear relationship (e.g. Fieldhouse et al., 2007; Verba & Nie, 1972), where turnout is relatively lower at the beginning of one's voting career and falls off again for the elderly (around 65 years and above). Recently, several studies have also tried to clarify the peculiar pattern of a so-called “first-time voter boost”. First time voters, usually 18- to 19-year-olds, vote more often than 20- to 21-year-olds who exhibit a markedly low turnout rate (see Bhatti & Hansen, 2012a; Bhatti et al., 2012; Konzelmann et al., 2012).

In this article we add empirical evidence to extend these findings for 16- and 17-year-old first-time voters. This article explores young voters' turnout for the case of Austria, which is the first of the EU member states and one of only a few countries in the world having a general voting age of 16 for all elections. We describe electoral turnout of young voters based upon electoral lists in two recent Austrian regional elections. Thus, for the first time this article presents evidence on turnout rates of 16- and 17-year-olds in a European country using official data. In particular, we will address two key questions: (1) Can the first-time voter boost also be observed for 16- and 17-year-old first-time voters? (2) Is the turnout rate of 16- and 17-year-olds higher than for other first-time voters?

The findings of this article will thus contribute to the larger debate on further lowering the voting age to 16, which is now debated in public in several European countries – among them Denmark, the UK and Norway.

## 2. Turnout of Young Voters

Why does age matter for turnout? It has been argued that the age between 18 and the mid-twenties is a critical phase in one's lifecycle and, thus the “political biography”. In young adulthood people have to make many important decisions that influence their whole life, such as deciding on an educational career, finding a job, choosing a partner, starting a family or moving to a new town. Therefore young people simply seem to be too preoccupied to worry about politics (Strate et al., 1989) and often exhibit lower political interest and civic duty (Blais et al., 2004; Blais & Rubenson, 2013). Moreover, several authors argued that social embeddedness is a driving factor in this context. While some young adults are still living at home, others have left their parents' household to start their “own life”. For instance, it has been shown that moving out of one's parents' home decreases turnout in the short run (Highton & Wolfinger, 2001), as the influence of parents decreases, while at the same time the influence of peers with weak voting habits increases (Bhatti & Hansen, 2012a). Mobility additionally increases the costs of voting, as young voters have to inform themselves about the political “supply” or orient themselves in new situations in life after secondary education (e.g. Blais, 2000). According to these findings, voting at the age of 18 or 19 seems to be easier and thus results in higher turnout rates than voting at the age of 20 to the mid-twenties. This pattern can be observed as the first-time voter boost (Bhatti & Hansen, 2012a; Bhatti et al., 2012; Konzelmann et al., 2012).

Anticipating these findings, Franklin (2004) pointed out that a first-time voting age of 18 was exceptionally disadvantageous. Given a four- or five-year electoral cycle, people experience their first election at 20 or 21 on the average, which – as pointed out above – is an inconvenient time to start one's voting career. The general decline of turnout in many Western democracies can partly be traced back to those cohorts that started with low turnout rates at their first elections when voting ages were lowered from 21 to 18 or 19 in many countries (Franklin, 2004; Franklin et al., 2004). Franklin's (2004) main argument in the debate on youth suffrage therefore was that one can expect *higher* levels of turnout among voters younger than 18 years, as life is still more “simple” and people are not yet that preoccupied positioning themselves in new social realities and they are primarily embedded in the social surroundings of family and school. At the same time, because of being strongly embedded in schools and families, young voters would hence “learn to vote” in a more sheltered environment. In particular, school attendance is seen as important factor in providing a stable social environment and also relevant information, which are, in general, the basis for political participation. This is of particular importance, bearing in mind the hypothesis that the first election leaves a footprint in one's voting biography and fosters voting as a habit (Dinas, 2012; Gerber et al., 2003; Plutzer, 2002). It is easier to repeat a learned behaviour that has already been performed than to override it with new behavioural patterns (Aldrich et al., 2011). Simply speaking, he who starts his electoral biography as a voter is likely to stay a voter. Hence, the lowering of voting age is also considered a possibility to restore higher levels of turnout in the long run (Franklin, 2004), assuming that younger first-time voters will maintain higher turnout rates.

However, the assumption that 16- and 17-year-olds will show a higher turnout level is not shared throughout the literature. The scientific controversy revolving around lowering the voting age has accumulated various arguments in favour or against such a reform. The arguments against foremost cover such concerns as the lack of political maturity, political interest and political knowledge of young voters which might lead to an uninformed vote choice (e.g. Bergh, 2013; Chan & Clayton, 2006; Electoral Commission, 2002, 2004; Hofer et al., 2008). Political maturation is assumed to increase when people grow older. Moreover, 16- and 17-year-olds are considered to be less interested and, hence, less likely to participate in an election than older first-time voters. Following this line, possible low turnout rates of 16- and 17-year-olds are thus used to strengthen the case against youth suffrage.

The arguments in favour, on the other hand, provide evidence that 16- and 17-year-olds are as ready to vote as are older voters in terms of political involvement (Hart & Atkins, 2011; Wagner et al., 2012; Wattenberg, 2008). Wagner et al. (2012) as well as Hart and Atkins (2011) argue that they are in no way inferior in their ability (e.g. quality of vote choice and knowledge) and willingness to participate in politics compared to other age groups. Still, regarding actual turnout rates empirical evidence is yet scarce. “Trial elections” that were held in Norwegian municipalities confirmed that turnout of 16- and 17-year-old enfranchised people was lower than overall turnout but higher than turnout of older first-time voters, which confirms Franklin's assumption. However, these findings do not necessarily apply to “real” elections, primarily because the municipalities participating in the trial elections volunteered to do so and were described as municipalities that were particularly engaged in youth politics (Bergh, 2013).

Our study will thus (a) explicitly test Franklin's (2004) hypothesis that turnout of the youngest voters is higher than for older first-time voters and (b) overcome some of the shortcomings of previous studies on youth turnout.

## 3. Case Selection: Austrian Regional Elections

Austria is known to be a country with traditionally high turnout rates. Even before the voting age was lowered, young Austrians were said to be comparable in terms of turnout to young people in countries such as Denmark, Sweden, Germany and the Netherlands (Milner, 2009). Some Austrian regions started lowering the voting age to 16 in regional and local elections in the first years of the millennium. Eventually, the federal electoral reform of 2007 included a general voting age of 16 for all elections, including federal elections, presidential elections and elections for the European Parliament, as well as referenda and all forms of plebiscites (Hofer et al., 2008). Meanwhile, the electoral law reform in Austria was accompanied by a bundle of measures for young voters, including an awareness-raising campaign and enhancing the status of civic and citizenship education in schools. It is safe to say that, at that time, first-time voters were also encouraged to participate in elections as schools were strongly engaged in preparing 16- and 17-year-olds for the federal elections of 2008, for instance (Schwarzer & Zeglovits, 2013).

So far, studies conducted in Austria found an increase in political interest among 16- and 17-year-olds after the time voting age was lowered (Zeglovits & Zandonella, 2013). As political interest can be regarded as an important motivational factor that increases participation (Verba et al., 1995), this finding would also suggest high turnout rates. In turn, the turnout *intention* of Austrian voters aged less than 18 in the weeks before the European Parliament elections of 2009 was lower than for voters that are aged 30 or older (Wagner et al., 2012). Consequently, this would suggest that turnout of 16- and 17-year-olds is, at least, lower than the average turnout rate.

Unfortunately, reliable data for the last federal elections in 2008 are not available. On the one hand, Austria is more restrictive in terms of data policy. Therefore no official registers of voters and factual participation in an election are available. On the other hand, surveys are usually biased by “overreporting” of voter turnout (e.g. Holbrook & Krosnick, 2010; for Austria see also Zeglovits & Kritzinger, 2013), and standard errors in surveys prevent one from detecting small group differences. This is why we examine turnout in two recent Austrian regional elections, where we were able to get access to electoral lists: the 2010 election in Vienna,^1^ the capital of Austria, and the 2012 election in Krems, a small sized town in Lower Austria. Choosing these elections has several advantages. First, both elections were already the second elections after lowering the voting age, so we do not expect much of an impact of “novelty” effects, as excitement should fade over time. Still, it should be mentioned that both elections are so-called “second order” elections. It is known that turnout rates and interest in these elections will be lower, in general. Moreover, one has to take into account that electoral turnout of young voters who have not yet developed a habit of voting is particularly low in second order elections (Franklin & Hobolt, 2011). So, we expect the importance to participate to be even lower and any differences in turnout to be even more noteworthy than in a first order election.

## 4. Data

For examining turnout among 16- and 17-year-olds we use electoral lists. Electoral lists include all eligible voters, that is, residents^2^ who are 16 years and older and have Austrian citizenship, and whether a voter did cast a ballot. The list has three possible outcomes: (1) voted, (2) registered as absentee voter, and (3) did not vote.

As turnout of persons who registered for absentee voting is not known, calculations were done twice when analysing turnout in the Vienna elections: First, we assume that all absentee voters (9.8% of eligible voters in the age group examined) did cast their vote (to be referred to as “maximum turnout”). Thus, maximum turnout will somewhat overestimate factual turnout. This is why we also tested our results against a more conservative estimator, which takes into account that the total number of voters in Vienna who registered for absentee voting was 162,039, while the total number of absentee votes was 129,332 or 79.8%. Since registered absentee voters can also cast their vote directly in the polling station, the figure only serves as a *lower* limit for turnout of absentee voters across all ages. Thus, the “low-rated turnout” estimator assumes that four out of five absentee voters cast their vote and will most likely underestimate turnout of young voters. It is approximately two percentage points lower than maximum turnout. However, substantive conclusions on Vienna presented below are not affected by the turnout estimate. The absentee voting phenomenon is, however, negligible in Krems as the total share of absentee voters was much lower (2.1%). This is why we also use the “maximum turnout” approach for Krems. As a robustness check we additionally ran all analyses again excluding absentee voters. Results do not change substantially.

The total age group of interest is 16- to 25-year-old voters. This allows for the comparison of all first-time voters and older (second-time) voters in the election, leading to the birth cohorts of 1985–1994 for the elections in Vienna 2010, and birth cohorts of 1987–1996 for the elections in Krems 2012.^3^ The age of the youngest birth cohort is limited by a due date for reaching age 16 before the election. However, as only the year of birth is included in the electoral lists, for some voters one cannot distinguish whether they were (older) first-time voters or (younger) second-time voters (see Table 1). When comparing 16- and 17-year-old first-time voters to older first-time voters, we will thus omit the oldest first-time voters (aged 21) from the analysis.

**Table 1.** Sample or population size by birth cohort

Year of birth	Age (approx.)	Year of first election	n
**Vienna**			Sample size
1994	16	2010	337
1993	17	2010	473
1992	18	2010	482
1991	19	2010	498
1990	20	2010	582
1989	21	2010 or 2005	617
1988	22	2005	560
1987	23	2005	638
1986	24	2005	619
1985	25	2005	605
Total	16–25		5,411
**Krems**			Population size
1996	16	2012	198
1995	17	2012	234
1994	18	2012	276
1993	19	2012	288
1992	20	2012	348
1991	21	2012 or 2007	397
1990	22	2007	420
1989	23	2007	398
1988	24	2007	406
1987	25	2007	339
Total	16–25		3,304

We had to limit ourselves to a sample procedure in Vienna^4^ (approx. 1,145,000 eligible residents). For this purpose we drew a two-stage stratified clustered sample within 59 polling stations, including n=5,411 eligible voters born between 1985 and 1994. All data analyses concerning Vienna take into account the clustered structure of the sample and standard errors are adjusted accordingly (Kish, 1995). In contrast, a full census was conducted in Krems, since the city is considerably smaller in size (approx. 23,000 eligible residents). For the city of Krems we examined all eligible voters born between 1987 and 1996 (n=3,304).

## 5. Results

**Figure 1.**
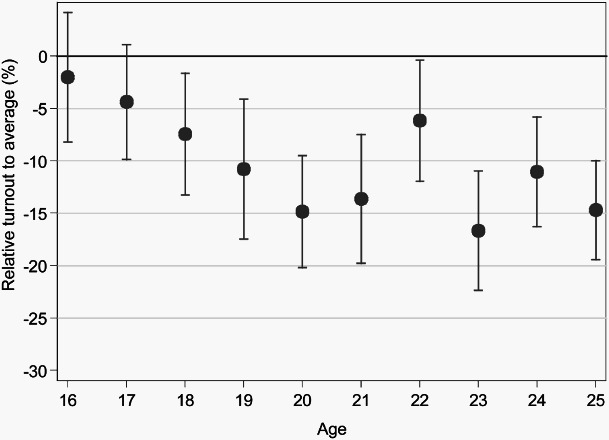
Turnout rate by age in Vienna using the maximum turnout estimator, relative to average turnout (67.6%).

*Note:* Graph for Vienna shows confidence intervals for the proportion (+/–1.96*S.E.); total n=5,411 within 59 clusters.

**Figure 2.**
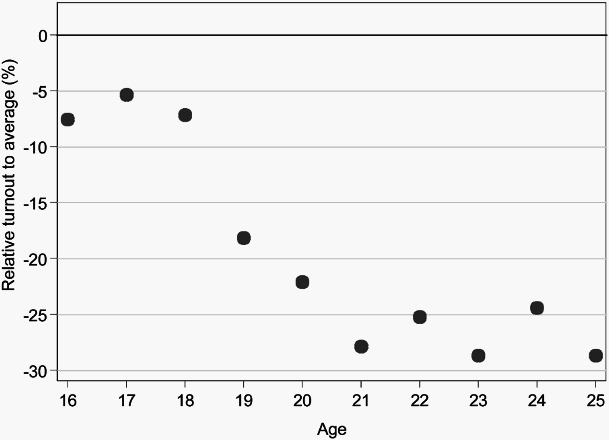
Turnout rate by age in Krems using the maximum turnout estimator, relative to average turnout (62.6%).

*Note:* Graph for Krems shows proportions of census data; total n=3,304.

Figure 1 and Figure 2 examine the levels of turnout by age. Figure 1 illustrates that, in Vienna, the turnout of 16- and 17-year-olds was not significantly lower than the overall turnout of 67.6%.^5^ This finding is robust, also if the low-rated turnout estimator is used (estimated turnout decreases by about 1.3 to 2.5 percentage points, depending on the share of absentee voters in each birth cohort). A similar phenomenon can be observed for the election in Krems (Figure 2): Turnout of 16- and 17-year-olds was only slightly lower than the overall turnout of 62.6%.^6^

With our data, the common finding that turnout of young voters is significantly and substantially lower than the overall turnout can be replicated, as long as only people aged 18 to 25 are concerned. Turnout of 16- and 17-year-olds rather resembles overall turnout.

Looking at the first-time voting boost in more detail, however, we observe that turnout *decreases* with increasing age from 16 to 20. In Vienna, this downturn is nearly linear: The older the first-time voter, the lower the turnout. In Krems, in contrast, 16- to 18-year-olds do not differ substantially in their turnout rate, but we see a sharp decline of turnout between age 18 and 19, similar to the decline described by Bhatti and Hansen (2012a) or Konzelmann et al. (2012). The first-time voting boost is thus found to be progressively less for older first-time voters.

In order to test Franklin's (2004) conjecture we next compare the overall turnout of 16- to 17-year-olds to the turnout of older first-time voters that are aged 18 to 20 (Tables 2 and 3). In fact, turnout of 16- to 17-year-olds in Vienna (Table 2) was estimated to be 64.2% and thus significantly and substantially higher than the turnout of 18–20-year-olds, which was 56.3%. In Krems turnout of 16- and 17-year-olds was 56.3% and substantially higher than turnout of older first-time voters (46.3%). In both towns we find that the share of the youngest first-time voters (16 to 17 years) participating in the election was higher than among older first-time voters (18 to 20 years). Our findings confirm Franklin's conjecture and Bergh's findings from the trial elections in Norway.

**Table 2.** Turnout of first-time voters in Vienna, 2010

	16–17 years	18–20 years
Voters	64.2	56.3
Non-voters	35.8	43.7
n	810	1,562
S.E. of proportion	+/*–*2.3	+/*–* 2.6
Corrected χ^2^, p=0.004		

*Note*: Maximum turnout estimator.

**Table 3.** Turnout of first-time voters in Krems, 2012

	16–17 years	18–20 years
Voters	56.3	46.3
Non-voters	43.7	53.7
n	432	912
χ^2^, p=0.001		

*Note*: Maximum turnout estimator, census data.

## 6. Discussion

When debating voting age, the case of Austria can provide empirical evidence on voting behaviour of enfranchised voters aged 16 to 17. In this article, we discussed electoral turnout in two Austrian regional (i.e. second order) elections, each of which was the second regional election after voting age was lowered to 16.

First, results from Vienna showed that turnout among 16- and 17-year-olds was not significantly lower than overall turnout, and also in Krems turnout was only somewhat lower than the average. The general trend that turnout of young people is by far lower than in the overall electorate cannot be applied to 16- and 17-year-old Austrian voters.

In both towns, turnout decreased with age for voters aged 18 to 21. Our study on 16- and 17-year-old voters thus confirms and extends previous evidence on the “first-time voting boost” phenomenon at the beginning of one's voting career, which has so far been described for countries having a voting age of 18 (Bhatti & Hansen, 2012a; Bhatti et al., 2012; Konzelmann et al., 2012). Second, Franklin's (2004) conjecture proved to be correct: electoral turnout of 16- and 17-year-olds was significantly *higher* than turnout of older first-time voters (18 to 20). Our study thus extends previous findings from Norwegian trial elections (Bergh, 2013) to a case study of “real” elections for a country having a general voting age of 16.

The results are particularly important in the debate about possible consequences of lowering the voting age to 16: our findings contradict the studies that assume low electoral participation of 16- and 17-year-olds because of lack of political interest (Chan & Clayton, 2006; Electoral Commission, 2004). Moreover, it has been argued that those who have not yet developed a habit to vote will especially abstain in second order elections (Franklin & Hobolt, 2011), such as the Austrian regional elections studied here. Thus, our findings of high turnout of young voters should also hold true in the case of first order national elections.

Of course, our results are limited. When interpreting the findings, one has to consider that elections take place in a certain societal context. The lowering of the voting age was accompanied by various measures, such as awareness-raising campaigns for the youngest eligible voters, in particular in the context of the federal election in 2008 (Schwarzer & Zeglovits, 2013, give an overview). It will take some time until we know if the effects observed here will continue, though accompanying measures cease to be continued. Future research will have to examine the short-term and long-term impact of the electoral reform on individual voting behaviour and aggregate turnout rates. With the data and design used here we simply cannot address whether higher turnout will actually leave a “footprint” in the youngest cohort's voting biography that continues in future elections. Finally, important information on individual covariates as well as the social embedding like family, schools and work, as well as spatial mobility, is missing. Further survey-based research is needed to disentangle the causes of participation or abstention of the youngest voters.

Online Appendix - Sampling procedure

## Notes

1 The City of Vienna lowered voting age for regional elections to 16 in 2005, that is, before the general reform.

2 Austrian citizens who reside in another country are eligible to vote in the municipality of their last residence in Austria.

3 Note that some voters in the cohort examined here may have participated in the general election in 2008. Though people acquire their habit of voting especially in first order national elections (see Franklin & Hobolt, 2011), we refrain from analysing the impact of this event on turnout rates of certain age groups, as we do not have the data to do so. Given the high turnout of the Vienna election, the findings of Franklin and Hobolt (2011) might not be perfectly applicable here.

4 A detailed description of the sampling procedure in Vienna is available in the online appendix (available on the publisher's website http://dx.doi.org/10.1080/17457289.2013.872652).

5 Note that the overall turnout rate in Vienna was similar to previous elections: 2005: 60.8%; 2001: 66.6%; 1996: 68.5%.

6 Note that the overall turnout rate in Krems was somewhat lower than in previous elections: 2007: 66.0%; 2002: 68.4%; 1997: 67.6%.
