# Supplementary material for: Are People More Inclined to Vote at 16 than at 18? Evidence for the First-Time Voting Boost Among 16- to 25-Year-Olds in Austria
Source: J Elect Public Opin Parties. 2014 Jan 8;24(3):351–61. doi: 10.1080/17457289.2013.872652 (PMC4864896; doi:10.1080/17457289.2013.872652)
Supplement: Online Appendix - Sampling procedure [file fbep_a_872652_sm5619.pdf]

Online Appendix: Sampling procedure Vienna 2010 regional elections

Eva Zeglovits, Julian Aichholzer

Supplementary to the research note "Are people more inclined to vote at 16 than at 18? Evidence for the first-time voting boost among 16- to 25-year-olds in Austria"

There is no access to electoral lists in Austria unless official approval by regional electoral authorities has been obtained. The electoral authorities of Vienna granted us access to the electoral lists of the regional elections of 2010. The sample was drawn for the polling stations, not for the voters. Within one polling station, all registered voters born in the years 1985 to 1994 were captured. Two sampling approaches were realized: first one was stratified random sampling of the polling station (Table A1), where the strata were formed by the size of each polling station (number of eligible voters). The number of polling stations to be sampled of each stratum was defined in accordance with the number of voters that were covered in each stratum. We had to use the number of total voters (of all ages), as there was no information on the relevant age group on the level of the entities, therefore the number of all voters was the only proxy available. 32 polling stations were sampled in order to reflect the voters in the different strata, as a compromise between high sample sizes for small margins of error and limits of resources. The second approach was theoretical sampling (Table A2). This time three strata were defined, each covering about 380,000 eligible voters. Out of each of these three strata, nine polling stations were selected: the three polling stations with the lowest turnout in the stratum, the three with the highest turnout in the stratum and the three polling stations with the central turnout. This makes another 27 polling stations in all. All analyses were run separately for the random sample and the theoretical sample, with one exception: for the ordered logistic regression model presented in Table 5, foreign residence cannot be used as a control variable in the random sampling approach, as there is not enough variance for this

variable. As data from both sampling strategies came up with the same results, everything was merged into one dataset later, covering 59 polling stations.

Table A1: Stratified clustered random sampling

stratum	size of polling station, eligible voters	mean number of eligible voters per polling station	number of polling stations in the stratum	number of eligible voters in the stratum	% of eligible voters in the stratum	number of polling stations selected
1	0 - 499	425,0	324	137.703	12.0%	4
2	500 - 599	551,6	421	232.220	20.3%	6
3	600 - 699	648,7	392	254.298	22.2%	7
4	700 - 799	745,1	334	248.857	21.7%	7
5	800 +	923,2	294	271.432	23.7%	8
total			1765	1.144.510	1	32

Table A2: Theoretical sampling

stratum	size of polling station, eligible voters	mean number of eligible voters per polling station	number of polling stations in the stratum	number of eligible voters in the stratum	% of eligible voters in the stratum	number of polling stations selected	
1	0 - 599	496.5	745	369.923	32,3%	9	3 low, 3 medium, 3 high
2	600 - 749	672.7	578	388.847	34,0%	9	3 low, 3 medium, 3 high
3	750+	872.7	442	385.740	33,7%	9	3 low, 3 medium, 3 high
total			1765	1.144.510	100%	27	
